# Personalized decision-making for aneurysm treatment of aneurysmal subarachnoid hemorrhage: development and validation of a clinical prediction tool

**DOI:** 10.1186/s12883-024-03546-x

**Published:** 2024-02-15

**Authors:** Jordi de Winkel, Bob Roozenbeek, Simone A. Dijkland, Ruben Dammers, Pieter-Jan van Doormaal, Mathieu van der Jagt, David van Klaveren, Diederik W. J. Dippel, Hester F. Lingsma

**Affiliations:** 1 Department of Neurology, Erasmus MC University Medical Center Rotterdam, 40 Doctor Molewaterplein, P.O. Box 2405, 3015 GD Rotterdam, Zuid-Holland The Netherlands; 2 Department of Public Health, Erasmus MC University Medical Center Rotterdam, Rotterdam, Zuid-Holland The Netherlands; 3 Department of Neurosurgery, Erasmus MC University Medical Center Rotterdam, Rotterdam, Zuid-Holland The Netherlands; 4 Department of Radiology and Nuclear Medicine, Erasmus MC University Medical Center Rotterdam, Rotterdam, Zuid-Holland The Netherlands; 5 Department of Intensive Care Adults, Erasmus MC University Medical Center Rotterdam, Rotterdam, Zuid-Holland The Netherlands

**Keywords:** Subarachnoid hemorrhage, Intracranial aneurysm, Prognosis, Personalized decision making

## Abstract

**Background:**

In patients with aneurysmal subarachnoid hemorrhage suitable for endovascular coiling and neurosurgical clip-reconstruction, the aneurysm treatment decision-making process could be improved by considering heterogeneity of treatment effect and durability of treatment. We aimed to develop and validate a tool to predict individualized treatment benefit of endovascular coiling compared to neurosurgical clip-reconstruction.

**Methods:**

We used randomized data (International Subarachnoid Aneurysm Trial, *n* = 2143) to develop models to predict 2-month functional outcome and to predict time-to-rebleed-or-retreatment. We modeled for heterogeneity of treatment effect by adding interaction terms of treatment with prespecified predictors and with baseline risk of the outcome. We predicted outcome with both treatments and calculated absolute treatment benefit. We described the patient characteristics of patients with ≥ 5% point difference in the predicted probability of favorable functional outcome (modified Rankin Score 0–2) and of no rebleed or retreatment within 10 years. Model performance was expressed with the *c*-statistic and calibration plots. We performed bootstrapping and leave-one-cluster-out cross-validation and pooled cluster-specific *c*-statistics with random effects meta-analysis.

**Results:**

The pooled *c*-statistics were 0.72 (95% CI: 0.69–0.75) for the prediction of 2-month favorable functional outcome and 0.67 (95% CI: 0.63–0.71) for prediction of no rebleed or retreatment within 10 years. We found no significant interaction between predictors and treatment. The average predicted benefit in favorable functional outcome was 6% (95% CI: 3–10%) in favor of coiling, but 11% (95% CI: 9–13%) for no rebleed or retreatment in favor of clip-reconstruction. 134 patients (6%), young and in favorable clinical condition, had negligible functional outcome benefit of coiling but had a ≥ 5% point benefit of clip-reconstruction in terms of durability of treatment.

**Conclusions:**

We show that young patients in favorable clinical condition and without extensive vasospasm have a negligible benefit in functional outcome of endovascular coiling – compared to neurosurgical clip-reconstruction – while at the same time having a substantially lower probability of retreatment or rebleeding from neurosurgical clip-reconstruction – compared to endovascular coiling. The SHARP prediction tool (https://sharpmodels.shinyapps.io/sharpmodels/) could support and incentivize a multidisciplinary discussion about aneurysm treatment decision-making by providing individualized treatment benefit estimates.

**Supplementary Information:**

The online version contains supplementary material available at 10.1186/s12883-024-03546-x.

## Background

As shown in multiple randomized controlled trials (RCTs), in aneurysmal subarachnoid hemorrhage (aSAH) patients that are equally eligible for endovascular coiling as for neurosurgical clip-reconstruction, endovascular coiling results in better functional outcome [1–4]. However, long-term follow-up revealed that patients who underwent neurosurgical clip-reconstruction had a lower rate of recurrent bleeding and retreatment of the target aneurysm and a higher degree of aneurysm obliteration [5, 6]. On average, this excess rebleeding and retreatment did not affect functional outcome [7].

Optimal aneurysm treatment is discussed in multidisciplinary teams where factors favoring neurosurgical clip-reconstruction or endovascular coiling are weighed. Examples of factors contributing to the choice for endovascular coiling are older age, worse clinical grade, vasospasm at presentation, and aneurysm location in the posterior circulation. On the other hand, neurosurgical clip-reconstruction is more likely to be performed in younger patients, patients with very small or very large (giant) aneurysms, wide-necked aneurysms, or middle cerebral artery aneurysms, or patients with concomitant intracerebral hemorrhage [8, 9]. When there is no preferred treatment, guidelines advise choosing endovascular coiling over neurosurgical clip-reconstruction [8, 9].

Average treatment effects found in RCTs apply to the study population as a whole and not to the individual patient [10]. Ideally, treatment decisions are based on the individualized treatment effects – or individualized treatment benefit. To estimate this, patient characteristics and their interaction with treatment have to be taken into account [10].

In the past, several studies developed models to predict outcome in aSAH patients, but none have focused on patient outcomes conditional on the choice of aneurysm treatment [11–15]. We hypothesize that the decision-making process could be improved by considering individualized treatment effects in terms of short-term functional outcome as well as the long-term durability of aneurysm treatment. Decision-making leading to a lower rate of rebleeding or retreatment while maintaining high probabilities of favorable functional outcome will benefit patients with aSAH. We aimed to develop and validate a prediction tool to predict benefit of endovascular coiling and neurosurgical clip-reconstruction in terms of short-term functional outcome and long-term durability of treatment in individual patients with aSAH.

## Methods

We previously published the study protocol and statistical analysis plan [16]. We used data from the randomized International Subarachnoid Aneurysm Trial (ISAT, *n* = 2143) [3]. We adhered to the Transparent Reporting of a multivariable prediction model for Individual Prognosis Or Diagnosis (TRIPOD) statement reporting guidelines (Additional file 1; Supplemental Methods 1) [17].

### Outcomes

We developed an ordinal regression model to predict short-term functional outcome (modified Rankin Scale; mRS) and a Cox regression model to predict time-to-rebleed-or-retreatment of the target aneurysm as an indicator of the durability of aneurysm treatment. We defined favorable functional outcome as an mRS score 0–2. In contrast with our initial statistical analysis plan, we used 2-month instead of 12-month mRS data. The reason for this change was to minimize the conditional dependence of the outcomes (the possibility that a rebleed or retreatment also affects the functional outcome or vice versa). Further, we used a Gaussian copula function to estimate the degree of residual correlation between the individual marginal probabilities of outcome (2-month favorable functional outcome and rebleed and retreatment within 10 years) in a joint model to determine if a separate interpretation of the probabilities is valid [18].

### Candidate predictors

We pre-specified potential predictors based on a clinically driven approach by literature review and clinical expertise. We considered age, time-to-aneurysm-treatment, World Federation of Neurological Surgeons (WFNS) grade, CT Fisher grade, vasospasm at presentation, aneurysm location, aneurysm lumen size, aneurysm neck size, and aneurysm treatment as potential predictors of outcome.

The WFNS grade is a clinical severity score to predict outcome after SAH ranging from I to V, good to poor clinical condition respectively [19]. The CT Fisher grade is an imaging-based score classifying the extent of subarachnoid hemorrhage and is used to predict intracranial vasospasm. The score ranges from 1, no SAH, to 4, presence of intraventricular or intracerebral clots [20]. We collapsed aneurysm locations located in the posterior circulation into one category: “posterior aneurysm location”. Aneurysm neck size was collected categorically in ISAT (dichotomized as ≤ 4 mm and > 4 mm) and could therefore not be analyzed continuously. To enhance the potential clinical application, we dichotomized vasospasm at presentation into “present” or “absent”. Time-to-aneurysm-treatment was defined as the time difference between randomization and treatment in days. Treatment in the first 24 h was scored as “zero days”. Time-to-aneurysm-treatment was truncated at the 95th percentile (21 days, *n* = 35). Missing time-to-aneurysm-treatment values were imputed with the value of the 95th percentile. Any patient who had a target aneurysm that was not treated was excluded from analysis in the Cox regression model (*n* = 35, (2%)). If a patient both had a rebleed and was retreated we classified this as a “rebleed”.

### Model development

The selection of predictors by univariable analysis for the multivariable model was based on a *p*-value of 0.20 to minimize overfitting and testimation bias [21]. We investigated non-linearity for continuous variables with restricted cubic splines. Based on clinical plausibility, we included (pre-specified) interactions of treatment assignment with age, aneurysm lumen size, aneurysm neck size, aneurysm location, and vasospasm at presentation. Further, we investigated the interaction between treatment assignment with the baseline risk of the outcome. The *p*-value for including a non-linear term was *p* < 0.05, and *p* < 0.01 for including an interaction term. Missing data were imputed with single imputation based on regression using the outcome, the candidate predictors with the addition of the patient’s sex. The proportion of missings was limited.

### Model performance

We expressed model performance in terms of discrimination and calibration with the *c*-statistic and calibration plots. Because the *c*-statistic for ordinal models has a more complicated interpretation than for models with a binary outcome we also calculated a *c*-statistic for the prediction of favorable functional outcome.

### Sensitivity analyses

We conducted two sensitivity analyses by re-running the model with separate outcomes (rebleed or retreatment) and with the 2-month and 12-month ordinal outcome (as originally intended) and evaluated the *c*-statistics.

### Validation

We used bootstrapping for internal validation to estimate the degree of optimism in the *c*-statistics of the final models. Further, we conducted leave-one-cluster-out cross-validation. Centers were assigned to form 4 clusters of approximately equal sample size. We pooled the resulting cluster-specific *c*-statistics with random effects meta-analysis and report prediction intervals (PI) [22].

### Benefit of treatment

For each patient, we predicted the probabilities of favorable functional outcome at 2 months and no rebleed or retreatment within 10 years follow-up with neurosurgical clip-reconstruction and with endovascular coiling. We derived treatment benefit for each patient – both in terms of favorable functional outcome at 2 months and no rebleed or retreatment within 10 years follow-up – by calculating the difference between outcome predictions of the two treatment modalities. For illustration, we described the patient characteristics of the population that had a large, ≥ 5% point, difference in treatment benefit of no rebleed or retreatment within 10 years with a negligible treatment benefit in terms of functional outcome. To assess the performance of the benefit predictions classical risk-prediction performance measures cannot be used. Comparing predicted benefit to observed benefit is not possible because the actual benefit of neurosurgical clip-reconstruction cannot be observed if a patient has been allocated to endovascular coiling (and vice versa). We used the *c*-for-benefit to assess the performance of the models in terms of benefit predictions [23, 24]. The *c*-for-benefit is defined as the proportion of all possible pairs of matched patient pairs with unequal observed benefit in which the patient pair receiving greater treatment benefit was also predicted to do so (Please see Additional file 1; Supplemental Table 1 for further explanation).

All statistical analyses were performed with R statistical software (version 4.1.1) using the *rms* (version 6.2.0), *Hmisc* (version 4.5.0), *survival* (version 3.3.1), *mice* (version 3.13.0), *metafor* (version 3.4.0), *CalibrationCurves* (version 0.1.2), *HTEPredictionMetrics* (version 0.1.1, https://github.com/CHMMaas/HTEPredictionMetrics) packages. To enhance the future application of the treatment benefit prediction models, we developed a web-based clinical prediction tool. This tool was developed with the *shiny* package (version 1.7.0).

## Results

We used the full cohort of 2143 patients for the development of the model predicting short-term functional outcome and 2108 patients for the development model predicting long-term time-to-retreatment-or-rebleed (Additional file 1; Supplemental Table 2). In the full cohort, the mean age was 51.7 (SD 11.6) years and 63% were women (*n* = 1345). Sixty-nine percent (*n* = 1467) had a favorable outcome at 2 months. At the longest follow-up, 133 (6%) patients were retreated and 75 patients (4%) rebled.

The short-term functional outcome model included the predictors’ age, aneurysm treatment, time-to-aneurysm-treatment, aneurysm lumen size, aneurysm location, WFNS grade, CT Fisher grade, and vasospasm at presentation (Table 1). We found no evidence for non-linearity or interaction with treatment. The long-term durability of treatment model included the predictors’ age, aneurysm lumen size, aneurysm location, and aneurysm treatment (Table 1). We did not find significant interaction with treatment. Age was added to the model as a non-linear term.

**Table 1:** Main effects of the models predicting functional outcome and long-term durability of treatment

**Variable**	**Ordinal model (*n* = 2143)**		**Cox model (*n* = 2108)**	
	**Univariable**	**Multivariable**	**Univariable**	**Multivariable**
	**cOR (95% CI)**	**cOR (95% CI)**^a^	**HR (95% CI)**	**HR (95% CI)**^b^
Treatment				
Endovascular	Ref	Ref	Ref	Ref
Neurosurgical	1.5 (1.3–1.7)	1.5 (1.3–1.7)	0.3 (0.2–0.4)	0.3 (0.2–0.4)
Time-to-aneurysm-treatment (days)	1.05 (1.03–1.08)	1.02 (1.00–1.04)	0.96 (0.93–1.00)	Not included
Age (per decade)				
Linear	1.2 (1.18–1.35)	1.20 (1.12–1.28)	0.88 (0.78–0.99)	NA
≤ 52		NA		0.7 (0.6–0.9)
> 52		NA		1.2 (0.9–1.5)
WFNS grade				
I	Ref	Ref	Ref	Not included
II	2.1 (1.8–2.5)	1.9 (1.6–2.3)	1.1 (0.8–1.5)	
III	3.5 (2.6–4.8)	2.5 (1.8–3.5)	0.6 (0.3–1.3)	
IV	7.6 (4.9–11.5)	6.6 (4.3–10.1)	1.3 (0.6–2.9)	
V	10.6 (4.8–23.7)	9.4 (4.1–21.5)	0.8 (0.1–5.6)	
CT Fisher grade				
1	Ref	Ref	Ref	Not included
2	1.0 (0.7–1.5)	1.2 (0.8–1.8)	1.1 (0.5–2.2)	
3	1.6 (1.1–2.2)	1.6 (1.1–2.3)	1.2 (0.6–2.4)	
4	2.6 (1.8–3.6)	1.9 (1.3–2.7)	1.2 (0.6–2.3)	
Aneurysm lumen size (mm)	1.05 (1.03–1.08)	1.05 (1.02–1.07)	1.06 (1.02–1.10)	1.06 (1.01–1.10)
Aneurysm neck size				
≤ 4 mm	Ref	Not included	Ref	Not included
> 4 mm	1.2 (1.0–1.4)		0.8 (0.6–1.1)	
Aneurysm location				
ACA	Ref	Ref	Ref	Ref
ACOM	1.0 (0.8–1.2)	0.9 (0.8–1.2)	1.6 (1.1–2.4)	1.6 (1.1–2.4)
ICA	1.2 (0.9–1.5)	1.3 (1.0–1.6)	1.5 (1.0–2.3)	1.4 (0.9–2.1)
MCA	1.0 (0.8–1.3)	0.9 (0.7–1.2)	1.3 (0.8–2.1)	1.2 (0.8–2.0)
PCOM	1.1 (0.8–1.4)	1.0 (0.8–1.4)	1.2 (0.7–2.1)	1.1 (0.6–1.9)
Posterior^c^	1.1 (0.7–1.8)	1.0 (0.6–1.7)	1.3 (0.5–3.3)	1.5 (0.6–3.7)
Vasospasm at presentation				
Absent	Ref	Ref	Ref	Not included
Present	1.6 (1.4–2.0)	1.2 (1.0–1.5)	0.9 (0.7–1.3)	

*Abbreviations: ACA* Anterior circulation artery, *ACOM* Anterior communicating artery, *CT* Computed tomography, *CI* Confidence interval, *HR* Hazard ratio, *ICA* Internal carotid artery, *MCA* Middle cerebral artery, *mm* milimeter, *PCOM* Posterior communicating artery, *Post *Posterior circulation, *ref* Reference category, *WFNS* World Federation of Neurological Surgeons grade

^a^An hazard ratio > 1 corresponds with a lower probability of no rebleed or retreatment

^b^A common odds ratio > 1 corresponds with a worse outcome

^c^Locations of aneurysms of the posterior circulation include: basilar artery, vertebral artery, superior cerebellar artery, anterior inferior cerebellar artery, posterior inferior cerebellar artery, and internal auditory artery

The internally validated *c*-statistics were 0.64 (95% CI 0.63–0.67) for the model with the ordinal outcome, 0.75 (95% CI 0.71–0.77) for the prediction of favorable functional outcome, and 0.68 (95% CI 0.65–0.72) for the durability of treatment model. The pooled *c*-statistics after leave-one-out cross-validation were 0.72 (PI 0.67–0.77) for the prediction of favorable outcome at 2 months and 0.67 (PI 0.63–0.71) for the prediction of no retreatment or rebleed within 10 years follow-up. The models were calibrated well over the range with the majority of the observations (Additional file 1: Supplemental Figs. 1A-D and Supplemental Figs. 2A-D). We found a limited residual correlation of 0.21 (95% CI 0.13–0.29) between time-to-rebleed-or-retreatment and favorable functional outcome at 2 months indicating that no further modeling is necessary for dependency.

### Sensitvity analyses

We did not observe any meaningful differences in the *c*-statistics in the sensitivity analysis by refitting the models with a separate outcome (rebleed or retreatment) or with a 2-month functional outcome versus a 12-month functional outcome.

### Benefit of treatment

**Fig. 1:**
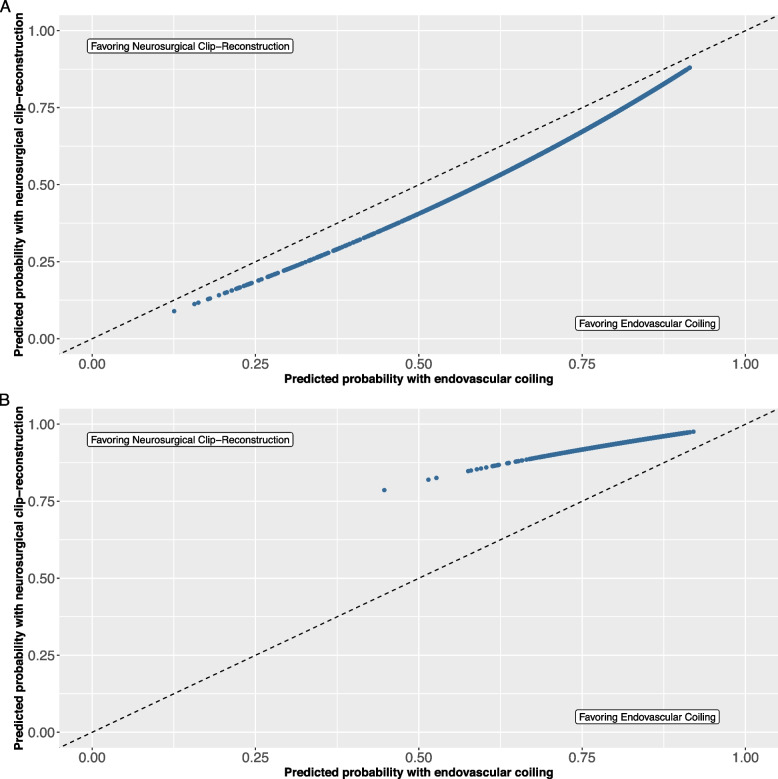
**A** Predicted probabilities of favorable functional outcome. **B** And durability of treatment Each dot represents an individual patient and his/her predicted probability of favorable functional outcome (mRS 0-2) or no rebleed or retreatment within 10 years with endovascular coiling and neurosurgical clip-reconstruction

We found an average difference in the predicted probability of the 2-month favorable functional outcome of 6% (95% CI 5–10, range 3–10%, Fig. 1A), favoring endovascular coiling. The *c*-for-benefit of this model was 0.57 (95% CI 0.53–0.61).

Further, we found an average difference in the predicted probability of no rebleed or retreatment within 10 years follow-up of 11% (95% CI 9–13, range 6–34, Fig. 1B), favoring neurosurgical clip-reconstruction. The *c*-for-benefit of this model was 0.57 (95% CI 0.50–0.63).

**Fig. 2:**
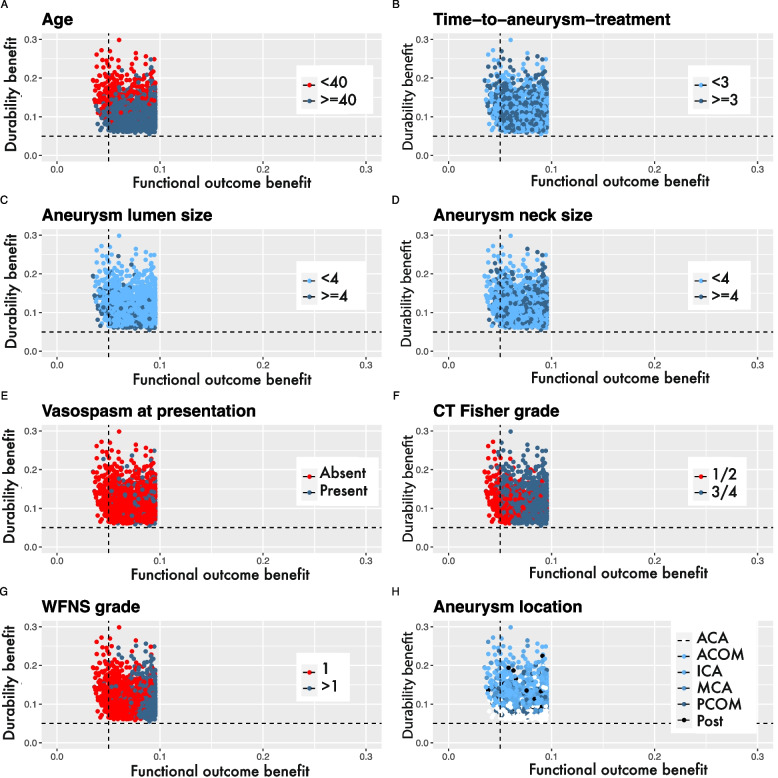
**A-H** Characteristics of population with large (≥ 5% point) benefit from neurosurgical clip-reconstruction Abbreviations: *ACA* = anterior circulation artery, *ACOM* = anterior communicating artery, *CT* = computed tomography, *ICA* = internal carotid artery, *MCA* = middle cerebral artery, *PCOM* = posterior communicating artery, *Post* = aneurysms of the posterior circulation, *WFNS* = World Federation of Neurological Surgeons. Locations of aneurysms of the posterior circulation include: basilar artery, vertebral artery, superior cerebellar artery, anterior inferior cerebellar artery, posterior inferior cerebellar artery, and internal auditory artery. Each dot corresponds to a patient in the International Subarachnoid Aneurysm Trial. The dashed lines indicate a ≥ 5% point difference in predicted probability of favorable functional (modified Rankin Scale 0–2) at 2 months or the difference in predicted probability of no rebleed or retreatment within a 10-year follow-up. Because no interactions were included in the models, every patient in the ISAT cohort had benefit of endovascular coiling in terms of functional outcome and benefit of clip-reconstruction in terms of durability of treatment. The values on the axis are calculated by subtracting the predicted probabilities of outcome. X-axis: P(mRS < 3) with endovascular coiling – P(mRS < 3) with neurosurgical clip-reconstruction, and on the y-axis P(No event within 10 years) with neurosurgical clip-reconstruction – P(No event within 10 years) with endovascular coiling. This creates four quadrants: Upper left quadrant: Population which should be considered for neurosurgical clip-reconstruction instead of endovascular coiling because of negligible benefit in functional outcome and ≥ 5% point difference in durability of treatment favoring neurosurgical clip-reconstruction. Lower left quadrant: Treat according to current guideline recommendations. No ≥ 5% point difference in treatment benefit with both outcomes. Upper right quadrant: Treat according to current guideline recommendations. A ≥ 5% point difference in treatment benefit for both outcomes. Lower right quadrant: Treat according to current guideline recommendations. Only ≥ 5% point difference in treatment benefit from endovascular coiling in terms of functional outcome

We identified 134 patients (6%) with a ≥ 5% point difference in the long-term durability of treatment favoring neurosurgical clip-reconstruction and with a negligible benefit of endovascular coiling in terms of short-term functional outcome. These patients had a mean age of 38 years (SD 9, Additional file 1: Supplemental Table 3) and had most often a WFNS grade I (97%), a CT Fisher grade 1–2 (83%), and no vasospasm at presentation (97%). Even though aneurysm lumen size, aneurysm location, and time-to-aneurysm-treatment were included in the models to predict outcome, they seem largely uninformative to identify patients that could benefit from choosing neurosurgical clip-reconstruction over endovascular coiling (Fig. 2A-H).

**Fig. 3:**
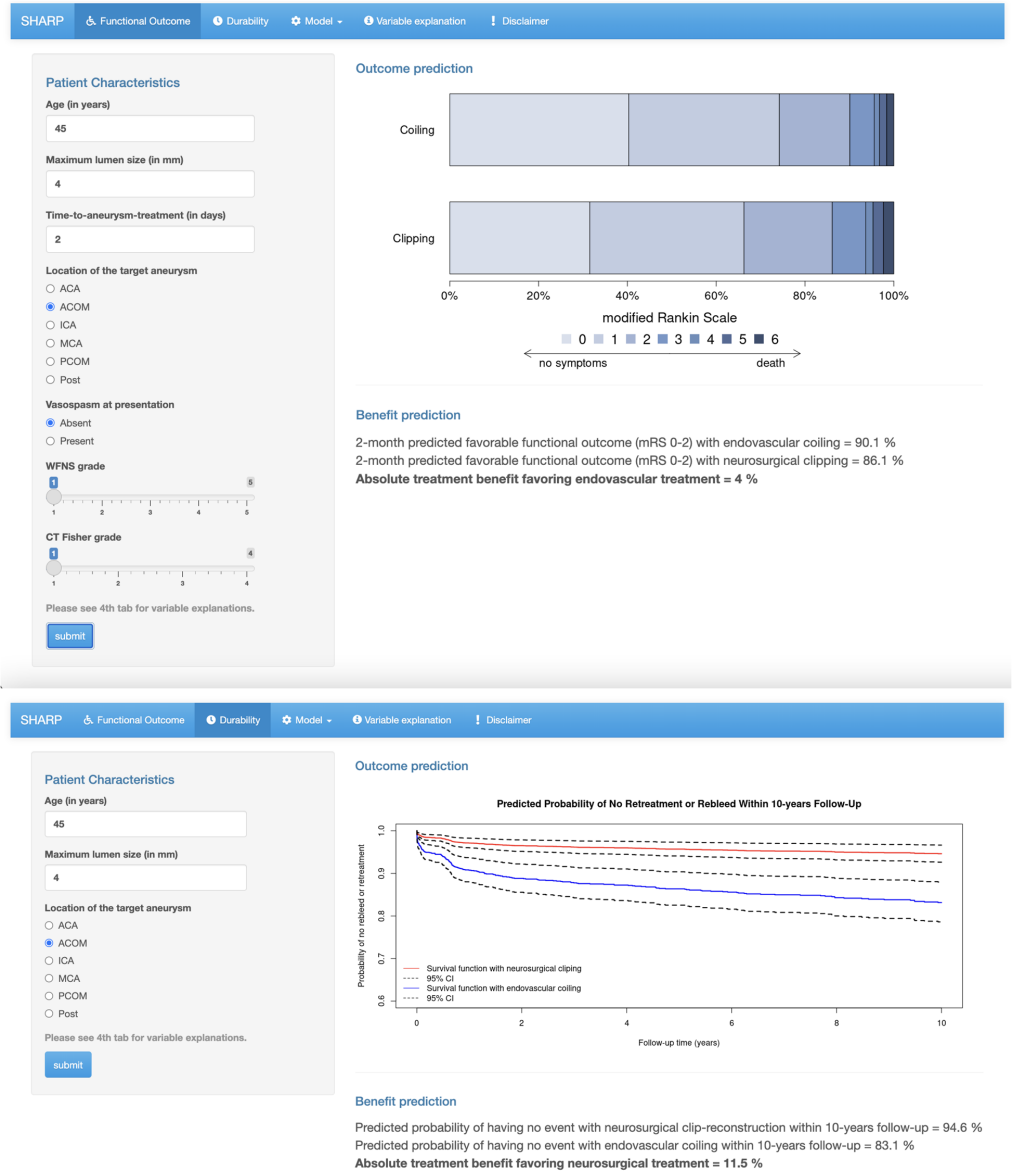
Visual representation of the web-based SHARP prediction tool The SHARP prediction tool is accessible via https://sharpmodels.shinyapps.io/sharpmodels/. Please note that the SHARP prediction tool is purely illustrative

Consider the following aSAH patient: a 45-year-old woman, presenting with a WFNS grade I and CT Fisher grade 1 because of a rupture of a 4-mm anterior communicating artery aneurysm, and without vasospasm at angiography. She is treated on the third day. According to the models this patient has predicted probability of 95% with neurosurgical clip-reconstruction and 83% with endovascular coiling of no rebleed or retreatment within 10 years follow-up, meaning an absolute treatment benefit of 12% point in favor of neurosurgical clip-reconstruction (Fig. 3). In terms of a 2-month favorable functional outcome, this patient has a predicted probability of 90% with endovascular coiling and 87% with neurosurgical clip-reconstruction, meaning a 3% point absolute benefit in favor of endovascular coiling.

### Model presentation

We developed the web-based Subarachnoid Hemorrhage Benefit of Treatment Prediction (SHARP) tool. This tool can be used to calculate absolute estimates of short-term favorable functional outcome and long-term durability of aneurysm treatment with neurosurgical clip-reconstruction and endovascular coiling. The SHARP prediction tool is accessible via https://sharpmodels.shinyapps.io/sharpmodels/. The regression formulas are presented in the prediction tool. Presently, this tool is purely illustrative meant to inform an incentivize a discussion about optimal aneurysm treatment and has not undergone a medical device regulation registration procedure.

## Discussion

We discovered that young patients who are in good clinical condition and have no extensive SAH or vasospasm at presentation benefit greatly from neurosurgical clip-reconstruction – compared to endovascular coiling – in terms of durability of treatment. At the same time, these patients did not benefit from endovascular coiling – compared to neurosurgical clip-reconstruction – because they had a high probability of favorable functional outcome regardless of the choice of treatment.

We did not find evidence for additive effects between treatment and the investigated predictors on functional outcome or durability of treatment. Because of this, the observed treatment benefit is purely risk-based. We applied a superior method to investigating heterogeneity of treatment effect than conventional subgroup analysis [2, 3, 25, 26]. First, because the tool’s risk-based benefit estimation is not hampered by the power issue of conventional subgroup analysis. Second, because a multivariable analysis is more reliable than a “one-variable-at-a-time” analysis.

Some decision problems require prediction tools to inform decision-making. For humans, it is impossible, to weigh a wide variety of patient characteristics and simultaneously consider non-linear effects. We developed models and the SHARP prediction tool that enables the prediction of individualized treatment benefit of endovascular coiling compared to neurosurgical clip-reconstruction accounting for short-term functional outcome and long-term durability of treatment for patients with aSAH. These estimates can be obtained relatively easily and could inform about the patient’s prognosis and support and incentivize a multidisciplinary discussion about optimal aneurysm treatment [27].

### Limitations

Some limitations have to be taken into account. We developed our models on data that was published nearly two decades ago. It is suggested that, due to the increased experience of interventionalists and the introduction of new devices, current endovascular and neurosurgical practices have improved. Subsequently, the average functional outcome after aSAH may have improved and retreatment and post-interventional rebleed rates may have declined [28]. This would affect the generalizability of our study.

However, the guideline recommendations that shape current clinical practice are limited by the same generalizability issue. This was recently illustrated by a study that established a large degree of practice variability in the (aneurysm) treatment of aSAH [29]. Until a new trial investigating the safety and efficacy of endovascular versus neurosurgical aneurysm treatment, reflecting contemporary practice is conducted, generalizability will remain unclear. It is unlikely that such a trial will take place in the foreseeable future. The current study, based on state-of-the-art modeling techniques, aimed to improve decision-making with the best data available and provides some evidence-based nuance to one-size-fits-all policy advised by current guidelines.

The lack of contemporary data also complicates validation. To assess the external validity of the benefit predictions only randomized data with long-term follow-up suffices [23]. The next best alternative to fully independent external validation is internal–external cross-validation [30]. With internal–external cross-validation, the validity of the model is assessed over clusters within the development data (e.g., by study, center, study period, or country). Because of the temporal and geographical clustering of the ISAT data, we were able to review model performance over multiple dimensions. Because in ISAT, the patients had to be equally amenable to neurosurgical clip-reconstruction as to endovascular coiling patient inclusion depended on the interpretation of local stroke specialists. This created a different case-mix across the participating sites that strengthens our belief in the validity of the model across multiple settings. It must be noted that ISAT included European aSAH patients exclusively, and that generalizability towards another ethnic population must be investigated in the future.

Another limitation is that the performance of the risk prediction models was only modest. This can be explained by several factors. In the first place, Harrell’s *c*-statistic for an ordinal outcome is a conservative measure and cannot be interpreted equally as for a model with a binary outcome. Concerning the Cox model, censoring may have affected the rank ordering (i.e. patients with a short survival time can also have a low predicted risk because of censoring) [31]. In the second place, a trial population with stringent selection criteria leads to decreased heterogeneity in the study population which makes it more difficult two discriminate between patients [32].

To inform decision-making, we only considered predictors that are available at baseline. Especially in the durability of treatment model, in the future, performance may be improved by adding predictors that are associated with incomplete occlusion of the aneurysm. Examples of these predictors could be complex aneurysm morphology, arterial branches springing from the aneurysm dome, or aneurysm neck size as a continuous variable [7]. Presently, it is difficult to appreciate the performance of the models in terms of benefit predictions. As is often the case, the *c*-for-benefit was low, but this measure lacks adjusted benchmarks and cannot be interpreted similarly as the Harrell’s *c*-statistic [23]. In any case, it is reasonable to assume that reliable outcomes estimates will most likely lead to reliable benefit estimates.

When applying the model in practice it will be difficult to weigh short-term functional outcome against the long-term durability of aneurysm treatment. Moreover, we used a composite durability outcome even though retreatment and rebleeding obviously have different clinical consequences. We hypothesized that retreatment and rebleeding are part of the same continuum. Untreated revascularization or remnants can lead to future rebleeding. The sensitivity analysis showed that both outcomes were associated with equal predictors with a comparable effect size. To illustrate the clinical impact, we illustrated a specific population that had a large (≥ 5% point difference) benefit in durability from neurosurgical clip-reconstruction without a benefit in functional outcome from endovascular coiling.

Nevertheless, we stress that short-term functional outcome and long-term durability of aneurysm treatment should not be valued equally. Given that the results of this study are complicated to extrapolate to current practice, the risk and benefit predictions should support rather than determine decision-making. Durability of treatment could be taken into consideration in the absence of (clinically relevant) benefit of functional outcome. Additionally, the individualized prognostic estimates can serve as a benchmark when new complex endovascular techniques are considered over established techniques. As a next step, we need a decision model that integrates the effects on both outcomes to translate treatment benefit to quality-adjusted life expectancy. Such a model could further facilitate (shared) personalized decision-making.

## Conclusions

In patients eligible for neurosurgical clip-reconstruction and endovascular coiling, on average, coiling leads to superior functional outcome. However, in some patients, only a negligible benefit of endovascular coiling over neurosurgical clip-reconstruction is expected, while at the same time, they do have much higher expected durability of treatment with neurosurgical clip-reconstruction compared to endovascular coiling. For these patients, best characterized as young and presenting in a favorable clinical condition, neurosurgical clip-reconstruction may be preferable. The SHARP prediction tool could support and incentivize a multidisciplinary discussion about personalized aneurysm treatment decision-making. Eventually, a new RCT is necessary to compare endovascular aneurysm treatment versus neurosurgical aneurysm treatment in a contemporary setting. Such a trial can be used to validate the proposed prediction tool.

### Supplementary Information

**Additional file 1:** **Supplemental Methods 1.** TRIPOD Checklist. **Supplemental Table 1. **Brief summary of the statistical concepts discussed in this paper. **Supplemental Table 2.** Baseline characteristics. **Supplemental Figures 1A-D.** Internal-external calibration plots of the model prediction short-term 2-month favorable functional outcome (modified Rankin Scale score 0-2). **Supplemental Figures 2A-D.** Internal-external calibration plots of the model predicting long-term within 10-year durability of treatment (no rebleed or retreatment). **Supplemental Table 3.** Baseline characteristics of the derivation cohort and the population that may benefit from neurosurgical clip-reconstruction.

## Data Availability

The data that support the findings of this study are available from the International Subarachnoid Aneurysm Trial (ISAT) investigators but restrictions apply to the availability of these data, which were used under license for the current study, and so are not publicly available. Data are however available from the authors upon reasonable request. After publication, the R code of the models and the SHARP prediction tool will be made available and accessible via: https://github.com/WinkelJordi/SHARP.

## Abbreviations

aSAH: Aneurysmal subarachnoid hemorrhage
CT: Computed tomography
ISAT: International subarachnoid aneurysm trial
mRS: modified Rankin Scale
PI: Prediction interval
RCT: Randomized controlled trial
SHARP: Subarachnoid hemorrhage benefit of treatment prediction
WFNS: World federation of neurological surgeons
