# Supplementary material for: Personalized decision-making for aneurysm treatment of aneurysmal subarachnoid hemorrhage: development and validation of a clinical prediction tool
Source: BMC Neurol. 2024 Feb 15;24:65. doi: 10.1186/s12883-024-03546-x (PMC10868110; doi:10.1186/s12883-024-03546-x)
Supplement: Supplementary file 1 — Additional file 1: Supplemental Methods 1. TRIPOD Checklist. Supplemental Table 1. Brief summary of the statistical concepts discussed in this paper. Supplemental Table 2. Baseline characteristics. Supplemental Figures 1A-D. Internal-external calibration plots of the model prediction short-term 2-month favorable functional outcome (modified Rankin Scale score 0-2). Supplemental Figures 2A-D. Internal-external calibration plots of the model predicting long-term within 10-year durability of treatment (no rebleed or retreatment). Supplemental Table 3. Baseline characteristics of the derivation cohort and the population that may benefit from neurosurgical clip-reconstruction. [file 12883_2024_3546_MOESM1_ESM.zip › Supplemental Material.docx]

**Supplemental Methods 1.** TRIPOD Checklist

| **Section/Topic** | **Item** |  | **Checklist Item** | **Page** |
| --- | --- | --- | --- | --- |
| **Title and abstract** | | | | |
| Title | 1 | D;V | Identify the study as developing and/or validating a multivariable prediction model, the target population, and the outcome to be predicted. | Title page |
| Abstract | 2 | D;V | Provide a summary of objectives, study design, setting, participants, sample size, predictors, outcome, statistical analysis, results, and conclusions. | Abstr. page |
| **Introduction** | | | | |
| Background and objectives | 3a | D;V | Explain the medical context (including whether diagnostic or prognostic) and rationale for developing or validating the multivariable prediction model, including references to existing models. | 5-6 |
|  | 3b | D;V | Specify the objectives, including whether the study describes the development or validation of the model or both. | 6 |
| **Methods** | | | | |
| Source of data | 4a | D;V | Describe the study design or source of data (e.g., randomized trial, cohort, or registry data), separately for the development and validation data sets, if applicable. | 6 |
|  | 4b | D;V | Specify the key study dates, including start of accrual; end of accrual; and, if applicable, end of follow-up. | Protocol paper |
| Participants | 5a | D;V | Specify key elements of the study setting (e.g., primary care, secondary care, general population) including number and location of centres. | Protocol paper |
|  | 5b | D;V | Describe eligibility criteria for participants. | Protocol paper |
|  | 5c | D;V | Give details of treatments received, if relevant. | Protocol paper |
| Outcome | 6a | D;V | Clearly define the outcome that is predicted by the prediction model, including how and when assessed. | 6 |
|  | 6b | D;V | Report any actions to blind assessment of the outcome to be predicted. | Protocol paper |
| Predictors | 7a | D;V | Clearly define all predictors used in developing or validating the multivariable prediction model, including how and when they were measured. | 7 |
|  | 7b | D;V | Report any actions to blind assessment of predictors for the outcome and other predictors. | Protocol paper |
| Sample size | 8 | D;V | Explain how the study size was arrived at. | 7 |
| Missing data | 9 | D;V | Describe how missing data were handled (e.g., complete-case analysis, single imputation, multiple imputation) with details of any imputation method. | 7&8 |
| Statistical analysis methods | 10a | D | Describe how predictors were handled in the analyses. | 8 |
|  | 10b | D | Specify type of model, all model-building procedures (including any predictor selection), and method for internal validation. | 7&8 |
|  | 10c | V | For validation, describe how the predictions were calculated. | 8-9 |
|  | 10d | D;V | Specify all measures used to assess model performance and, if relevant, to compare multiple models. | 8 |
|  | 10e | V | Describe any model updating (e.g., recalibration) arising from the validation, if done. | NA |
| Risk groups | 11 | D;V | Provide details on how risk groups were created, if done. | 9 |
| Development vs. validation | 12 | V | For validation, identify any differences from the development data in setting, eligibility criteria, outcome, and predictors. | 9-10 |
| **Results** | | | | |
| Participants | 13a | D;V | Describe the flow of participants through the study, including the number of participants with and without the outcome and, if applicable, a summary of the follow-up time. A diagram may be helpful. | 7/Suppl |
|  | 13b | D;V | Describe the characteristics of the participants (basic demographics, clinical features, available predictors), including the number of participants with missing data for predictors and outcome. | Prot paper/Suppl |
|  | 13c | V | For validation, show a comparison with the development data of the distribution of important variables (demographics, predictors and outcome). | NA |
| Model development | 14a | D | Specify the number of participants and outcome events in each analysis. | 10 |
|  | 14b | D | If done, report the unadjusted association between each candidate predictor and outcome. | NR |
| Model specification | 15a | D | Present the full prediction model to allow predictions for individuals (i.e., all regression coefficients, and model intercept or baseline survival at a given time point). | 13 |
|  | 15b | D | Explain how to the use the prediction model. | 15-18 |
| Model performance | 16 | D;V | Report performance measures (with CIs) for the prediction model. | 10-11 |
| Model-updating | 17 | V | If done, report the results from any model updating (i.e., model specification, model performance). | NA |
| **Discussion** | | | | |
| Limitations | 18 | D;V | Discuss any limitations of the study (such as nonrepresentative sample, few events per predictor, missing data). | 13-14 |
| Interpretation | 19a | V | For validation, discuss the results with reference to performance in the development data, and any other validation data. | NA |
|  | 19b | D;V | Give an overall interpretation of the results, considering objectives, limitations, results from similar studies, and other relevant evidence. | 12-15 |
| Implications | 20 | D;V | Discuss the potential clinical use of the model and implications for future research. | 16 |
| **Other information** | | | | |
| Supplemental information | 21 | D;V | Provide information about the availability of Supplemental resources, such as study protocol, Web calculator, and data sets. | 6, 13 |
| Funding | 22 | D;V | Give the source of funding and the role of the funders for the present study. | Back matter |

**Supplemental Table 1.** Brief summary of the statistical concepts discussed in this paper.

| **Concept** | **Meaning** |
| --- | --- |
| *Performance of risk predictions* | |
| Discrimination | The ability to discriminate between high-risk patients and low-risk patients. Does the model accurately identify those that experience the outcome? |
| Harrell’s *c*-statistic | Used to assess discriminative performance for binary and time-to-event outcomes. The *c*-statistic is the proportion of all possible pairs of observations discordant on the outcome (i.e., one with the outcome and one without), in which the subject with the outcome had a higher predicted probability than the one without the outcome. |
| Calibration | The agreement between the predicted risk and the observed risk. How accurate is the risk prediction? Calibration can be measured by the difference between the predicted and observed outcomes in the groups per decile predicted risk. |
| Intercept | The intercept is the ratio between expected and observed outcomes. Ideally, the intercept has a value of 0, whereas a negative value means an overestimation of the predicted risk to the observed risk, and a positive value is an underestimation of the predicted risk to the observed risk. |
| Slope | The beta value of the calibration model. Evaluates the spread of the estimated risks and ideally have a value of 1. A value below 1 means the estimated risks are too extreme and a value above 1 means the estimated risks are too moderate. |
| Eavg | When fitting a lowess-estimated calibration curve through the calibration groups, the Eavg is the average absolute calibration error between the diagonal and the lowess-estimated calibration curve. |
| *Performance of benefit predictions* | |
| *c*-for-benefit | Measure for the discriminative ability of models predicting individualized treatment effect. The *c*-for-benefit corresponds to the probability that from two randomly chosen matched patient pairs with unequal observed treatment effect, the pair with greater observed treatment effect also has a higher predicted treatment effect.  In simpler words: you compare the averaged predicted benefit with treatment A and treatment B in two matched patient pairs with the observed benefit.  The observed benefit is derived from subtracting the observed outcomes of the matched patient pairs. With a binary outcome (e.g., dead or alive) all possible benefit/harm combinations are alive-alive, alive-dead, dead-alive, and dead-dead. These combinations translate to no benefit, benefit/harm, and no benefit, or in mathematical terms 0, +1 or -1, and 0.  The predicted benefit is derived by subtracting the predicted probabilities of the outcome of both treatments based on the model.  When concordant, the difference in predicted benefit between the matched pairs (more beneficial) should match the difference in observed benefit (+1). If not, the matched pair is discordant. If there is no difference in the observed benefit then the pair is uninformative. This information is used to create a rank-order-based discrimination measure equal to the conventional *c*-statistic.  The *c*-for-benefit is defined as the proportion of all possible pairs of matched patient pairs with unequal observed benefit in which the patient pair receiving greater treatment benefit was predicted to do so. The *c*-for-benefit thus represents the probability that from two randomly chosen matched patient pairs with unequal observed benefit, the pair with greater observed benefit also has a higher predicted benefit. |

**Supplemental Table 2.** Baseline characteristics.

| **Variable** | **Ordinal model cohort (*n* = 2143)** | **Cox model cohort (*n* = 2108)** |
| --- | --- | --- |
| Age (years) – No. (%) | 2143 (100) | 2108 (100) |
| Mean (SD) | 52 (11.6) | 52 (11.5) |
| Sex – No. (%) | 2143 (100) | 2108 (100) |
| Female | 1345 (63) | 1323 (63) |
| WFNS grade – No. (%) | 2112 (99) | 1978 (94) |
| I | 1335 (63) | 1321 (64) |
| II | 549 (26) | 540 (26) |
| III | 134 (6) | 131 (6) |
| IV | 74 (4) | 69 (3) |
| V | 20 (1) | 17 (1) |
| CT Fisher grade – No. (%) | 2129 (99) | 2094 (99) |
| 1 | 114 (5) | 114 (5) |
| 2 | 360 (17) | 357 (17) |
| 3 | 905 (42) | 892 (43) |
| 4 | 753 (35) | 731 (35) |
| Severity of vasospasm at presentation – No. (%) | 2143 (100) | 2108 (100) |
| Absent | 1694 (79) | 1678 (80) |
| Present | 449 (21) | 430 (20) |
| Aneurysm lumen size (mm) – median (range) | 5 (1-31) | 5 (1-31) |
| Aneurysm neck size | 2095 (98) | 2094 (99) |
| >4mm – No. (%) | 580 (28) | 580 (28) |
| Aneurysm location – No. (%) | 2143 (100) | 2108 (100) |
| Anterior cerebral artery | 528 (25) | 514 (24) |
| Anterior communicating artery | 556 (26) | 550 (26) |
| Internal carotid artery | 490 (23) | 484 (23) |
| Middle cerebral artery | 303 (14) | 299 (14) |
| Posterior communicating artery | 207 (10) | 203 (10) |
| Posterior circulation aneurysms^*^ | 59 (3) | 58 (3) |
| Allocated treatment – No. (%) | 2143 (100) | 2108 (100) |
| Endovascular | 1073 (50) | 1065 (50) |
| Neurosurgical | 1070 (50) | 1043 (50) |
| Time-to-aneurysm-treatment (days) – median (IQR)^†^ | 3 (2-6) | 3 (2-6) |

**Abbreviations:** CT = computed tomography, IQR = interquartile range, mm = millimeter, SD = standard deviation, WFNS = World Federation of Neurological Surgeons.

^*^ Posterior circulation aneurysms include the basilar artery, vertebral artery, superior cerebellar artery, anterior inferior cerebellar artery, posterior inferior cerebellar artery, and internal auditory artery.

^†^ Time-to-aneurysm-treatment was truncated at 14 days. In the ordinal model, missing time-to-aneurysm-treatment was imputed with the mean. In the Cox model, any patient that has not received aneurysm treatment will be imputed with 14 days.

**Supplemental Figures 1A-D.** Internal-external calibration plots of the model prediction short-term 2-month favorable functional outcome (modified Rankin Scale score 0-2).

We created the validation datasets by binning the 46 centers into 4 subsets of approximately equal sample size.

**Supplemental Figures 2A-D.** Internal-external calibration plots of the model predicting long-term within 10-year durability of treatment (no rebleed or retreatment).

We created the validation datasets by binning the 46 centers into 4 subsets of approximately equal sample size.

**Supplemental Table 3.** Baseline characteristics of the derivation cohort and the population that may benefit from neurosurgical clip-reconstruction.

| **Variable** | **Derivation cohort (*n* = 2143)** | **Population that may benefit from neurosurgical clip-reconstruction (*n* = 134)** |
| --- | --- | --- |
| Age (years) – mean (SD) | 52 (11.6) | 38 (9) |
| WFNS grade – No. (%) |  |  |
| I | 1335 (62) | 130 (97) |
| II | 549 (26) | 0 |
| III | 134 (6) | 0 |
| IV | 74 (3) | 1 (1) |
| V | 20 (1) | 3 (2) |
| Fisher grade – No. (%) |  |  |
| 1 | 114 (5) | 37 (28) |
| 2 | 360 (17) | 74 (55) |
| 3 | 902 (42) | 19 (14) |
| 4 | 753 (35) | 4 (3) |
| Severity of vasospasm at presentation – No. (%) |  |  |
| Absent | 1694 (79) | 130 (97) |
| Present | 449 (21) | 4 (3) |
| Aneurysm lumen size (mm) – median (IQR) | 5 (4-7) | 5 (4-8) |
| Aneurysm neck size >4mm – No. (%) | 580 (27) | 30 (22) |
| Aneurysm location – No. (%) |  |  |
| Internal carotid artery | 490 (23) | 15 (11) |
| Anterior cerebral artery | 528 (25) | 28 (21) |
| Middle cerebral artery | 303 (14) | 28 (21) |
| Anterior communicating artery | 556 (26) | 48 (36) |
| Posterior communicating artery | 207 (10) | 12 (9) |
| Other posterior circulation aneurysms^*^ | 59 (3) | 3 (2) |
| Time-to-aneurysm-treatment (days) – median (IQR)^†^ | 3 (2-6) | 3 (2-5) |

**Abbreviations:** CT = computed tomography, IQR = interquartile range, mm = millimeter, mRS = modified Rankin Scale, SD = standard deviation, WFNS = World Federation of Neurological Surgeons grade

^*^ Locations of aneurysms of the posterior circulation include the basilar artery, vertebral artery, superior cerebellar artery, anterior inferior cerebellar artery, posterior inferior cerebellar artery, and internal auditory artery.

^†^ Time-to-aneurysm-treatment is truncated at 14 days. In the ordinal model, missing time-to-aneurysm-treatment will be imputed with the mean. In the Cox model, any patient that has not received aneurysm treatment will be imputed with 14 days.
