# Supplementary figures and images for: Personalized decision-making for aneurysm treatment of aneurysmal subarachnoid hemorrhage: development and validation of a clinical prediction tool
Source: BMC Neurol. 2024 Feb 15;24:65. doi: 10.1186/s12883-024-03546-x (PMC10868110; doi:10.1186/s12883-024-03546-x)

A

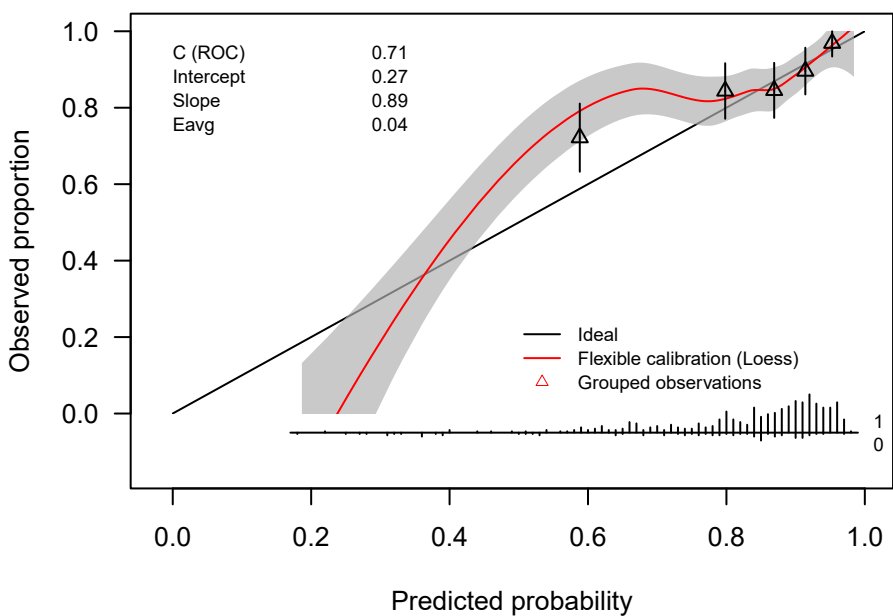

B

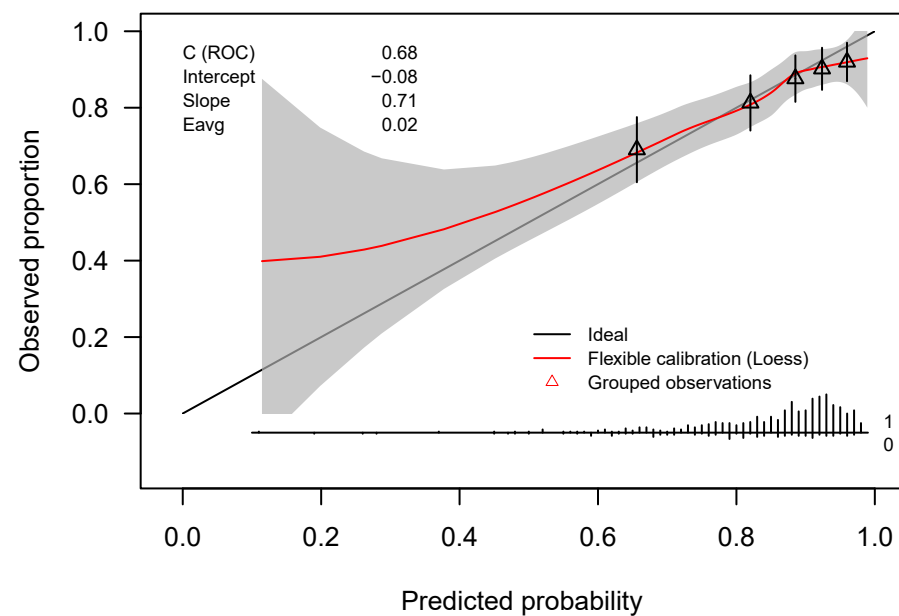

C

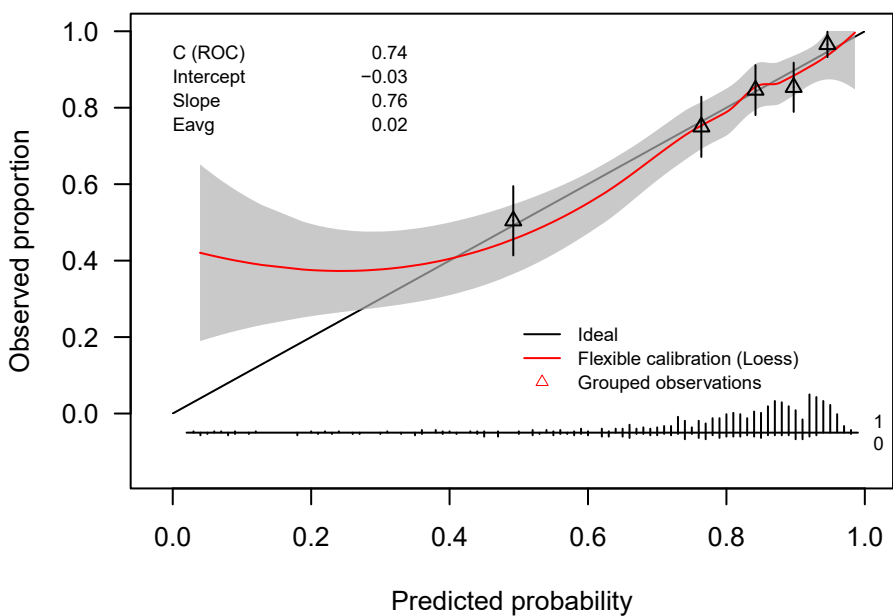

D

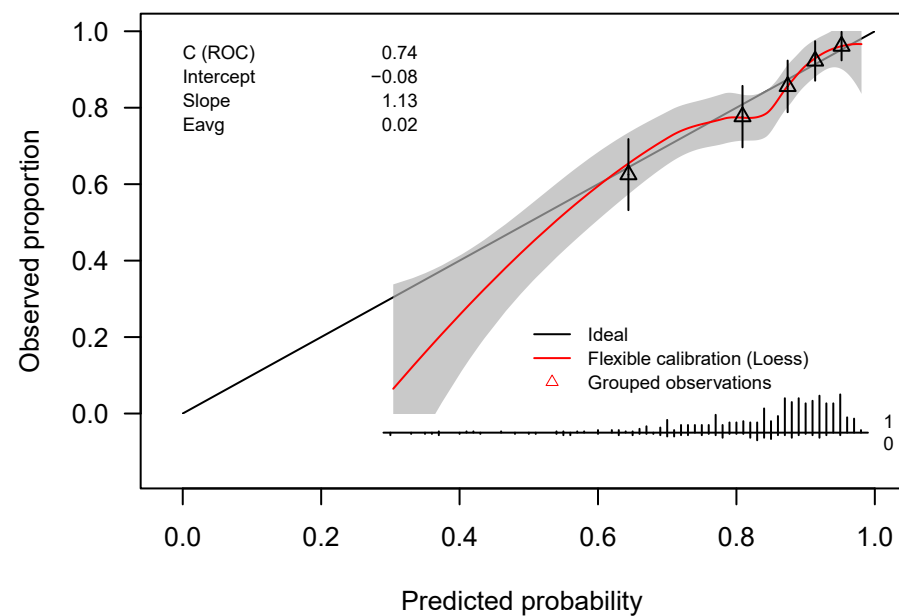

Supplement: Supplementary file 1 — Additional file 1: Supplemental Methods 1. TRIPOD Checklist. Supplemental Table 1. Brief summary of the statistical concepts discussed in this paper. Supplemental Table 2. Baseline characteristics. Supplemental Figures 1A-D. Internal-external calibration plots of the model prediction short-term 2-month favorable functional outcome (modified Rankin Scale score 0-2). Supplemental Figures 2A-D. Internal-external calibration plots of the model predicting long-term within 10-year durability of treatment (no rebleed or retreatment). Supplemental Table 3. Baseline characteristics of the derivation cohort and the population that may benefit from neurosurgical clip-reconstruction. [file 12883_2024_3546_MOESM1_ESM.zip › Supplementary Figures 1A-D.pdf]

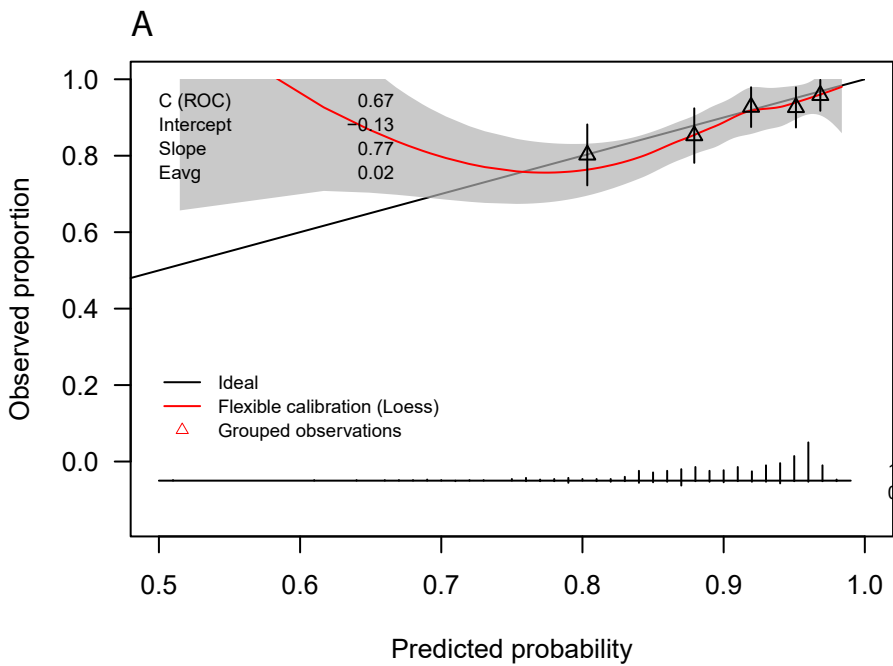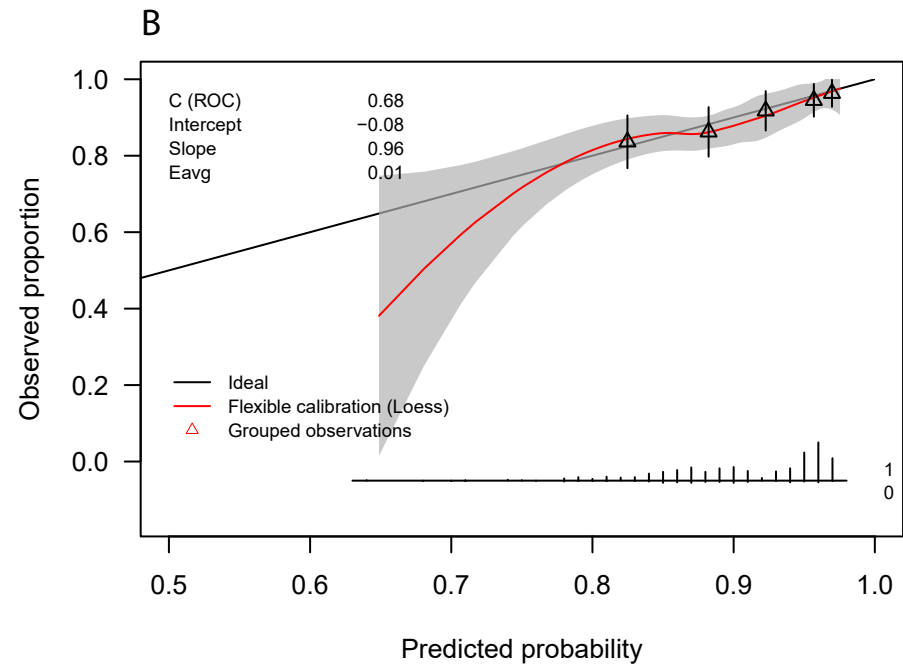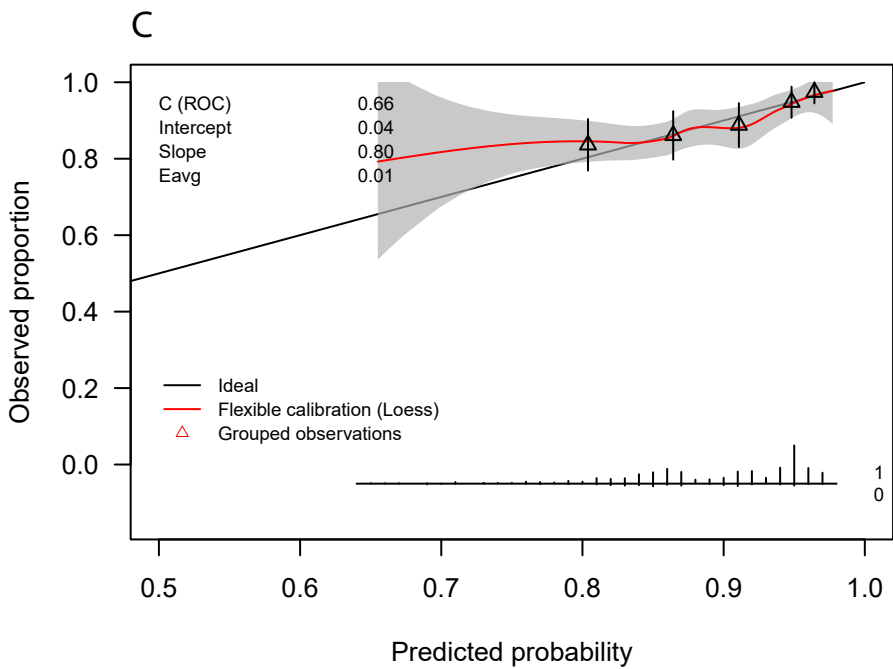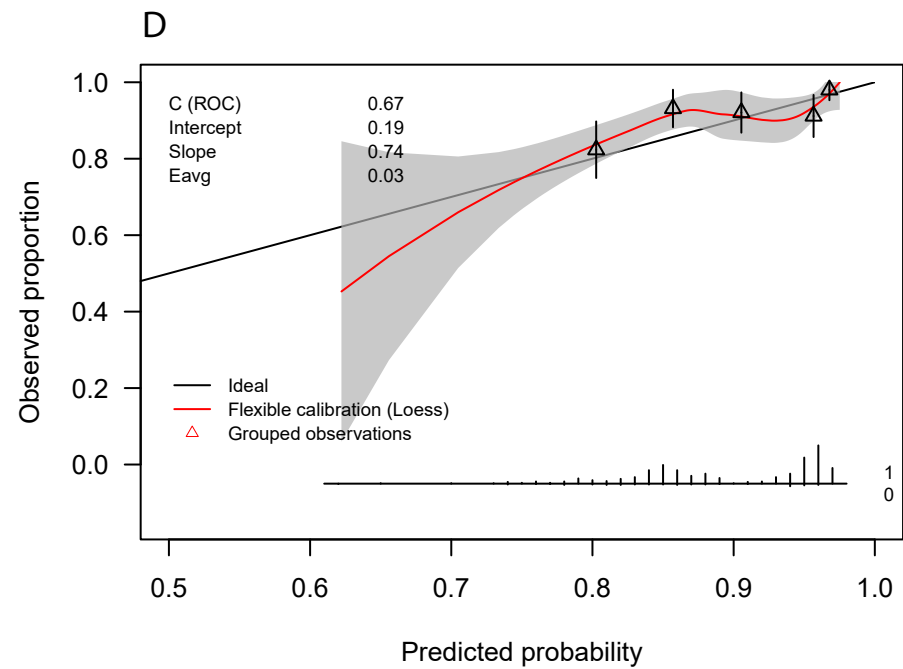

Supplement: Supplementary file 1 — Additional file 1: Supplemental Methods 1. TRIPOD Checklist. Supplemental Table 1. Brief summary of the statistical concepts discussed in this paper. Supplemental Table 2. Baseline characteristics. Supplemental Figures 1A-D. Internal-external calibration plots of the model prediction short-term 2-month favorable functional outcome (modified Rankin Scale score 0-2). Supplemental Figures 2A-D. Internal-external calibration plots of the model predicting long-term within 10-year durability of treatment (no rebleed or retreatment). Supplemental Table 3. Baseline characteristics of the derivation cohort and the population that may benefit from neurosurgical clip-reconstruction. [file 12883_2024_3546_MOESM1_ESM.zip › Supplementary Figures 2A-D.pdf]
